# Myasthenic crisis treated in a Chinese neurological intensive care unit: clinical features, mortality, outcomes, and predictors of survival

**DOI:** 10.1186/s12883-019-1384-5

**Published:** 2019-07-19

**Authors:** Fan Liu, Qiong Wang, Xueping Chen

**Affiliations:** 1 Department of Neurology, West China Hospital, Sichuan University, 610041, Guoxuexiang #37, Chengdu, Sichuan China; 2 Department of Nursing, West China Hospital of Stomatology, Sichuan University, Chengdu, Sichuan China; 3 Neurological Intensive Care Unit, West China Hospital, Sichuan University, Chengdu, Sichuan China

**Keywords:** Myasthenic crisis, Intensive care unit, Clinical characteristics, Management, Outcomes, Survival predictor

## Abstract

**Background:**

Myasthenic crisis (MC) often requires admission to an intensive care unit (ICU).

**Methods:**

We retrospectively investigated 113 consecutive patients with first MC admitted to the neurological ICU. Patients’ demographic, clinical and other characteristics were examined, as well as therapeutic interventions, mortality and functional outcome.

**Results:**

MC patients at first onset admitted to neurological ICU had a mortality rate of 18.6%. PCO_2_ level before intubation and score on Myasthenia Gravis–Activities of Daily Living (MG-ADL) scale at MC onset correlated with duration of ventilation and length of ICU stay. Compared with patients with good functional outcome, patients with intermediate or poor functional outcome were older at first MC onset, had lower pH and PO_2_, and had higher PCO_2_ before intubation. Multivariate logistic analysis identified pre-intubation PCO_2_ level as an independent predictor of survival. Cox regression showed that age at first MC onset requiring ICU management was the factor which significantly influenced the mortality.

**Conclusions:**

Our results suggest that PCO_2_ before intubation and MG-ADL score at MC onset may be useful indicators of more severe disease likely to require extensive respiratory support and ICU management. Higher pre-intubation PCO_2_ indicates chronic respiratory acidosis that can increase risk of severe disability and death, especially in patients with older age at first MC onset.

**Electronic supplementary material:**

The online version of this article (10.1186/s12883-019-1384-5) contains supplementary material, which is available to authorized users.

## Background

Myasthenia gravis (MG) is a neuromuscular disorder, characterized by muscle weakness and easy fatigability upon exertion. It is caused by the action of antibodies against proteins in the neuromuscular junction, and the most common autoantibody is the anti-acetylcholine receptor (AChR) antibody. This antibody reduces the number of postsynaptic acetylcholine receptors available on the end plate of the skeletal muscle. The second most prevalent antibody recognizes muscle-specific kinase (MuSK); this autoantibody is found in up to 70% of AChR-negative MG patients [1].

One of the most serious complications of MG is myasthenic crisis (MC), characterized by increased weakness of respiratory muscles that leads to acute respiratory failure requiring mechanical ventilation [2]. MC occurs in 10–20% of MG patients over the course of their disease [3–6]. While associated with mortality rates as high as 50–80% in the 1960s, MC is now often reported to be fatal in fewer than 5% of cases as a result of the development of intensive care techniques [2, 6, 7].

We undertook a retrospective study of MC patients with an exacerbation of MG admitted to the neurological intensive care unit (ICU) to understand more about the clinical characteristics, management and outcomes of patients with first MC who were requiring ICU management, We also performed regression analysis to identify baseline clinical and other factors that might predict survival for those MC patients.

## Methods

### Patients

The MG patients at our hospital were followed up since July 2008, including a cohort of 2023 MG patients. Patients had been diagnosed with MG because they showed two or more of the following clinical features: clinical evidence of muscle weakness and easy fatigability, significant decremental response on repetitive nerve stimulation, presence of anti-AChR or anti-MuSK antibodies, or objective clinical response to the neostigmine test [3]. Other diseases that mimic MG must be excluded, including Lambert-Eaton myasthenic syndrome, peripheral neuropathy, myopathies and motor neuron disease. MC was defined as respiratory failure from muscle weakness requiring mechanical ventilation with intubation or noninvasive ventilatory support (continuous or bi-level positive airway pressure) [3]. Patients were intubated if clinical assessment indicated compromised respiratory effort documented by decreased patient comfort with either single breath count less than 10, respiratory rate more than 30 per minute, decreased chest expansion, paradoxical diaphragmatic movements or arterial blood gas showing drop in oxygen saturation. Normal oxygen saturation does not exclude MC, and oxygen saturation drops late in neuromuscular respiratory failure. If life-threatening hypoxemia (PaO_2_ < 60 mmHg) occurs, and cannot be improved with supplemental oxygen, intubation is required. Furthermore, general criteria for intubation also included impaired swallowing mechanism leading to ineffective cough and nasal voice, inability to clear secretions. MG patients intubated for respiratory failure due to acute respiratory distress syndrome, congestive heart failure, and hypoxic-ischemic coma were excluded since in those cases respiratory muscle weakness was not a contributing factor for intubation. Therefore, we excluded one patient who was intubated for overwhelming asthma and one patient who was intubated following a cardiorespiratory arrest. Post-thymectomy crises were also excluded. In this retrospective study, patients admitted to the neurological ICU with first MC onset were investigated. MC patients managed only on the general ward were not included in the study. The decision to mechanically ventilate was taken on a case-by-case basis according to the patient’s respiratory condition and with the consent of patients or relatives. Death of MC patients occurring at ICU and as well as at the residence were recorded. The period of evaluation of the patients was until May 2017.

### Data collection

The following demographic data were recorded for each patient: gender, age of MG onset, age at first MC onset requiring ICU management, comorbidities, presence of anti-AChR or -MuSK antibodies, and history of thymoma, thymectomy, and other autoimmune diseases. In addition, the following information was also collected: MG duration before MC, score on Myasthenia Gravis–Activities of Daily Living (MG-ADL) scale at disease onset, score on the Myasthenia Gravis Foundation of America (MGFA) scale at ICU admission, length of hospital stay, length of ICU stay, and duration of ventilator use. Primary precipitating factors were identified whenever possible; these included infection, surgery, medication, aspiration, pregnancy, and stressor. Possible secondary precipitating factors were also identified. Data was collected on the last time of arterial blood gas measurements before intubation, including white blood cell count (WBC), Ph, PO_2_, and PCO_2_. Functional outcomes were assessed using the MGFA postintervention status every 3 months after discharge from the ICU. All included patients had follow-up duration of 12 months after discharged from ICU. In the present study, patients with different functional outcome were considered to experience good, intermediate, and poor prognosis. Good outcome was defined as achievement of complete stable remission (CSR), pharmacologic remission (PR) or minimal manifestations (MM). A status of improvement (IM) was categorized as intermediate outcome. Unchanged (U), worse (W), exacerbation (E) and died (D) were classified as poor outcome. CSR indicates no fatigable muscle weakness of MG for at least one year and being free of medication for MG. PR shows those MG patients taking some form of drug for MG excluding cholinesterase inhibitors, but with the same clinical criteria as for CSR. MM indicates MG patient who has no symptoms of functional limitations but has some weakness that is only detectable by careful examination. IM indicates a sustained substantial reduction in MG medications or substantial decrease in pretreatment clinical manifestations.

### Statistical analysis

Descriptive statistics were used to evaluate clinical features. Data for continuous variables were compared between groups using the independent *t* test, while data for categorical variables were compared using the chi-squared or Fisher’s exact tests. Correlation analyses were performed using Spearman’s rank correlation test. The following variables were tested for their potential association with outcome: age of first MC onset requiring ICU management, arterial blood gas values, WBC, MG-ADL at MC onset, duration of ventilation and hospitalization, and ICU stay. Predictors of poor outcome were determined using logistic regression, followed by Cox regression to identify significant independent predictors. All analyses were performed using SPSS 18.0 (IBM, Chicago, IL) and a threshold of significance of *p* <  0.05.

## Results

### Demographic and clinical information

During the study period, 113 patients (39 males, 74 females) suffered first MC onset requiring ICU management were admitted to our neurological ICU (Table 1). The rate of first MC was 5.6%. Mean age at MG onset was 39.5 years (range, 15.2 to 73.6 years). Mean duration from MG onset to first MC onset requiring ICU management was 24.1 months (range, 1.2 to 110.5). Fifty-eight percent of patients experienced their first MC within one year after symptom onset. Mean age at first MC onset requiring ICU management was 40.5 years (range, 17.4 to 75.8), and 79 patients (20 males, 58 females) suffered first MC onset while younger than 50 (mean age at onset, 32.1). The remaining 36 patients (19 males, 17 females) experienced first MC onset when they were older than 50 (mean age at onset, 60.4).

**Table 1:** Baseline demographic and clinical data of patients with first MC onset

Parameter	value
Age at MG onset (yr)	39.5 ± 16.7
Age at first MC onset (yr)	40.5 ± 16.1
Duration of MG before first MC (mo)	24.1 ± 25.3
Neostigmine Test (positive/negative/not tested)	95/13/5
RNS (positive/negative/not tested)	83/17/13
Anti-AChR auto-antibodies (positive/negative/not tested)	62/30/21
Precipitant (Infection/Medication/ Pregnancy/ Stressor/Hypokalemia without precipitant)	58/18/3/1/1/32

Abbreviations: *MC* myasthenic crisis, *mo* month(s), *yr.* year(s), *RNS* repetitive nerve stimulation

All patients met at least one positive diagnostic test recognized in the inclusion criteria. The neostigmine test showed the highest overall sensitivity (88.0%, 95/108 positive, 5 not tested), followed by the repetitive stimulation test (83.0%, 83/100 positive, 13 not tested), and the anti-AChR autoantibody test (67.4%, 62/92 positive, 21 not tested) (Table 1).

### Precipitants of MC

Infection was the most frequent precipitant of MC, occurring in 51.3% of cases (58/113). This took the form of lower respiratory tract infection in 47 patients and upper respiratory infection in 11 patients. In addition, 8 of these 59 patients experienced both infection and hypokalemia. Precipitating factors due to medication occurred in 18 patients (15.9%); 11 patients among them developed MC due to failure to comply with treatment, and MC in 7 patients was aggravated by high-dose steroid therapy. These 7 patients received oral steroid before crises, but acute deterioration following initiation of intra-venous methylprednisolone 1000 mg/day treatment precipitated the ICU admission, and they showed symptoms of severe weakness of the respiratory and/or bulbar muscles and inability to maintain adequate ventilation. Three patients had MC during pregnancy (2.7%), one patient developed MC with emotional upset after losing his family member (0.9%), and one patient had MC after severe hypokalemia (0.9%). No obvious precipitant was identified in the remaining 32 patients (28.3%) (Table 1).

### Respiratory status, mechanical ventilation, and ICU stay

Included patients spent a mean of 12.2 days (range, 1 to 55) in the ICU and a mean of 26.3 days (range, 3 to 81) in the hospital overall. All patients received mechanical ventilation, for a mean duration of 190.3 h (range, 4 to 925). Respiratory status of all patients was evaluated prior to intubation, the last time of blood gas analysis before intubation indicated mean pH of 7.4 (range, 7.1 to 7.6), mean PO_2_ of 105.3 mmHg (range, 56 to 220), and mean PCO_2_ of 48.4 mmHg (range, 18.3 to 93.1). Leukocytosis was seen in 107 patients, and mean white blood cell count before intubation was 16.5 × 10^9^/L (range, 8.0 × 10^9^ to 25.8 × 10^9^) (Table 2). At the time of MC onset, 38 (33.6%) had mild symptoms (IIB), 62 patients (554.9%) had moderate symptoms (MGFA IIIB), and 11 (9. 7%) had severe symptoms (IVB) (Table 2). In addition, 28 patients (24.8%) received tracheostomies (MGFA V) at a mean of 10.5 days after presentation. The MGFA status of most patients progressively improved during follow-up. Mean MG-ADL score at first MC onset was 16.9, and it correlated positively with PCO_2_ before intubation (*p* = 0.004). Several factors were assessed for possible association with duration of mechanical ventilation, hospital stay and ICU stay. Both PCO_2_ before incubation and MG-ADL score at first MC onset correlated positively with duration of ventilation and ICU stay. Longer duration of mechanical ventilation correlated positively with longer stay in the ICU and in the hospital (Table 3).

**Table 2:** Laboratory tests, disease characteristics and outcomes of patients with first MC onset

Parameter	value
Arterial blood gas before intubation	
pH	7.4 ± 0.1
PO_*2*_ (mmHg)	105.3 ± 43.6
PCO_*2*_ (mmHg)	48.8 ± 16.8
Blood test	
WBC (10^*9*^*/L)*	16.5 ± 6.2
Clinical severity	
MGFA (IIB/IIIB/IVB)	38/62/11
MG-ADL scale score	16.9 ± 4.4
Duration on ventilation (hours)	190.3 ± 221.6
Duration of hospitalization (days)	26.5 ± 15.3
ICU stay (days)	12.3 ± 13.1

Abbreviations: *ICU* intensive care unit, *MC* myasthenic crisis, *MG-ADL* Myasthenia Gravis–Activities of Daily Living scale, *MGFA* Myasthenia Gravis Foundation of America scale, *mRS* modified Rankin scale, *WBC* white blood cell count

**Table 3:** Factors associated with duration on ventilation, duration of hospitalization, and ICU stay

	duration on ventilation		ICU stay	
	r_s_	*P* value	r_s_	*P* value
Age at first MC onset	0.342	0.063	0.232	0.168
pH before intubation	- 0.173	0.372	- 0.145	0.487
PO2 before intubation	- 0.267	0.144	- 0.213	0.239
PCO2 before intubation	0.319	**0.043**	0.413	**0.025**
MG-ADL at first MC onset	0.541	**0.002**	0.753	**< 0.0001**
duration on ventilation	/	/	0.841	**< 0.0001**

Abbreviation: *ICU* intensive care unit; Significant values are highlighted in bold characters

### Treatment

Prior to admission to ICU, most patients received acetylcholinesterase inhibitors; and pyridostigmine bromide was given to 101 MC patients (89.4%) before intubation. Four patients continued on a decreased dose after establishing mechanical ventilator support. Seventy-five patients were on oral prednisolone or intravenous methylprednisolone prior to admission to ICU (66.4%). During stay in ICU, 104 patients were given steroids (92.0%). Specifically, intra-venous methylprednisolone 1000 mg/day was administered for 3–5 days, followed by oral prednisolone (1 mg/kg/day). During ICU staying, intravenous immunoglobulin (IVIG) therapy was given to 76 patients (67.3%), 67 of whom received steroid pulse IVIG combination therapy. Four patients received the combination of corticosteroids, IVIG, and plasma exchange. The remaining patients were unable to receive steroids or IVIG or plasma exchange because of hemodynamic instability or severe sepsis or the family’s economic hardship. Other oral immunosuppressants such as tacrolimus were given to 12 patients (10.4%).

### Functional outcome, mortality, and comorbidities

For the prognosis, 87 patients (77.0%) showed good outcome, 5 (4.4%) had intermediate and 21 (18.6%) had poor outcome. Eighteen patients died in the ICU; all these fatalities resulted from severe comorbidities that had kept them bed-ridden for a long time: pneumonia and respiratory failure (*n* = 7); bacteremia sepsis (*n* = 4); and uremia (*n* = 3) and heart failure (n = 3), followed by respiratory arrest (*n* = 1). Three patients died after discharge from the ICU: one patient’s condition improved upon admission to the ICU, but she was not fully recovered, and she died 22 days later due to acute exacerbation of pneumonia after discharging from hospital. Two patients stopped treatment and requested early discharge because of financial problems. The twenty-one patients who died were significantly older than those who survived (*p* = 0.0009). Table 4 summarized the characteristics of all the included MC patients categorized to two outcome groups, those with good outcome (*n* = 87) and those with intermediate or poor outcome (*n* = 26). The mean age of first MC onset requiring ICU management was older in the intermediate or poor outcome group, the difference was statistically significant. Patients with intermediate or poor outcome had significantly lower pH and PO_2_ as well as significantly higher PCO_2_ in the last time of blood gas analysis before intubation, compared to patients with good outcome. In contrast; the two groups did not differ in WBC count or in the duration of ventilation, hospitalization, or ICU stay. Comparison of comorbidities between patients with good outcome and those with intermediate or poor outcome showed that there was no significant difference in comorbidities among patients with different outcomes (Table 4).

**Table 4:** Comparison of patients showing good or poor outcome*

	Good outcome (*n* = 87)	Intermediate or poor outcome (*n* = 26)	*P* value
Age at first MC onset	32.2 ± 7.7	54.2 ± 15.9	**< 0.0001**
pH	7.4 ± 0.6	7.3 ± 0.13	**0.0004**
PO2	116.9 ± 42.7	80.2 ± 32.5	**0.0396**
PCO2	37.9 ± 7.9	58.6 ± 20.8	**0.0005**
WBC	14.5 ± 4.6	16.1 ± 5.4	0.4557
Duration on ventilation, h	168.0 ± 233.9	241.8 ± 268.8	0.2853
Duration of hospitalization, d	21.3 ± 15.2	28.3 ± 17.7	0.5334
ICU stay, d	10.2 ± 16.3	17.2 ± 14.3	0.1707
Comorbidities	32.2 ± 7.7	54.2 ± 16.0	**< 0.0001**
Hypertension	24	12	0.0565
DM	10	6	0.1961
Hyperlipidemia	9	4	0.4920
Heart disease	3	1	1.0000
COPD	3	2	0.3245
Stroke	1	1	0.4088
Cancers	4	1	1.0000
SLE	3	0	0.1707
AID	2	0	1.0000
Hepatitis	5	2	0.6600
Hyperthyroidism	2	0	1.0000

Abbreviations: *ICU* intensive care unit, *WBC* white blood cell count, *DM* diabetic mellitus, *SLE* Systemic lupus erythematosus, *AID* autoimmune disease, *COPD* Chronic obstructive pulmonary disease; Significant values are highlighted in bold characters

Univariate analysis using the log rank test identified the following four variables as significantly associated with survival: age at first MC onset requiring ICU management, gender, PO_2_, and PCO_2_ (Table 5). Older age of first MC requiring ICU management, male gender, lower PO_2_, and higher PCO_2_ were associated with higher mortality risk. However, multivariate logistic regression only identified pre-intubation PCO_2_ as an independent factor associated with survival. Higher pre-intubation PCO_2_ was associated with higher mortality risk. Cox regression identified that age at first MC onset requiring ICU management was the key factor which significantly influenced the mortality among the variables examined (*p* = 0.031).

**Table 5:** Predictors of death

	Exp (Coef)	95% CI	*P* value
Univariate			
Age at first MC onset	1.135	1.040 to 1.238	**0.004**
Gender	8.500	1.247 to 57.931	**0.029**
PO_*2*_	0.838	0.698 to 0.011	0.059
PCO_*2*_	1.181	1.026 to 1.358	**0.020**
WBC	1.068	0.910 to 1.253	0.422
MG-ADL at first MC onset	1.176	0.949 to 1.459	0.139
Duration of ventilation	1.001	0.998 to 1.004	0.522
Duration of hospitalization	1.006	0.958 to1.056	0.824
ICU stay	1.011	0.951 to1.075	0.725
Multivariate			
PCO_*2*_	1.147	1.010 to 1.303	**0.034**

Abbreviations: *ICU* intensive care unit, *MG-ADL* Myasthenia Gravis-Activities of Daily Living scale, *WBC* white blood cell count; Significant values are highlighted in bold characters

## Discussion

MC is a potentially life-threatening complication in patients with MG, but the mortality rate has fallen dramatically over the past 60 years. The introduction of the neurological ICU has substantially improved early recognition of MC, identification of its precipitating factors and respiratory management of patients. The present work may help further improve the early recognition and care of patients who suffer MC by providing a picture of clinical characteristics (Additional file 1: Table S1) and even suggestions of baseline factors that may help predict survival.

Mean age at first MC onset requiring ICU management was 40.5 years. However, the median age at first MC onset was 55 years in a US study [3]. One possible explanation for this discrepancy is ethnicity; other explanations include the differences in sample size, environmental factors and other population factors. In the present study, we found that first MC affecting people younger than 50 years affected women disproportionately, most of whom were aged 20–50; in contrast, first MC affecting people older than 50 did not show gender bias. These results are consistent with other studies [3, 6, 8, 9]. The average interval from onset of MG to first MC requiring ICU management was 24.06 months in our cohort, much longer than the 8 months reported in another study [3]. Our results are consistent with recent reports of a median interval from onset to crisis of 3 years [9] and mean duration of MG prior to ICU admission of 3.8 years [10]. A longer interval from MG onset to first MC probably reflects recent improvements in recognition of the disease, management of respiratory and bulbar conditions, and greater access to newer treatment modalities. Just over half our patients experienced their first MC within one year of symptom onset, consistent with a study showing that MC typically occurs within the first 2 years after MG diagnosis [11].

While MG diagnosis in Europe and North America is most often supported using the tensilon test, the neostigmine test is used more often in China. In our study, the neostigmine test showed overall sensitivity of 88.0%, a little bit lower compared to the 96.8% reported by another study in China [12]. These results validate the important role of this test for MG diagnosis in China. The proportion of patients in our cohort who took the repetitive stimulation test and gave a positive result was 83.00%, higher than the 77.4% reported in a cohort of 1108 Chinese MG patients [12], and higher than the 75.9% reported in an Italian cohort [13]. The higher rate of positive results on the repetitive stimulation test in our study may reflect the fact that we included all MC patients admitted to the ICU during the study period, none of whom had ocular MG. Sixty-two patients in 92 cases were positive for anti-AChR antibodies. This may underestimate the real prevalence of such antibodies, since this test is not routine in China because of resource limitations.

Infection, especially lower respiratory tract infection, was the most common identifiable precipitant of MC, followed by medication, and inadequate treatment/drug withdrawal. Other studies have also identified respiratory tract infection as the most frequent cause of MC, accounting for about half of crises resulting in ICU admission [9, 10, 14]. Failure to comply with treatment or drug withdrawal was a frequent cause of MC accounting for 11 patients out of 113 in our study. Initial treatment with steroid led to exacerbation of MG in 30–50% of patients and decompensation in patients with MC, whereas 9–18% of them develop MC [15]. In the present study, 7 patients out of 113 develop MC due to high-dose steroid therapy. Therefore, initiation of high-dose steroid should occur in a hospital setting, where the respiratory function can be monitored [15]. Predictors of exacerbation from steroid include older age, bulbar symptoms, and lower score on Myasthenia Severity Scale [15–17]. Our study showed pregnancy as a trigger of MC being responsible for crisis in 3 out of 113 patients, and study reported that pregnancy can aggravate MG in 33% of the MG cases [5, 18, 19]. We suggest a detailed review of systems when the disease is getting worse, with attention to infectious sources, respiratory symptoms, and drug exposures (12). Physicians must pay careful attention to respiratory rate, difficulty with phonation, a quiet voice, weak neck muscles, work of breathing, and oxygenation. If the patient demonstrates vital capacity (VC) < 10–20 mL/kg or negative inspiratory force (NIF) < − 20 to − 30 cm H2O, diagnosis of MC is considered. However, these values are not derived from studies on patients with MG, but rather from studies in patients with GBS. We recommend that physicians should focus on the respiratory status of the patient, and trends in these symptoms, rather than relying on absolute numbers of VC or NIF. We also identified higher MG-ADL score at MC onset as a potential indicator that ICU care will be needed. Indeed, MG-ADL score > 18 points at MC has been reported to predict the need for ICU management with 75% sensitivity and 77.8% specificity [14]. Surprisingly, we detected severe hypercarbia in our cohort before intubation (mean PCO_2_, 48.78 mmHg). Since MG-ADL score at MC onset correlated positively with PCO_2_ before intubation, respiratory status may be tightly associated with MC symptoms, and hypercarbia may affect daily activities of patients with MC. Mean duration of ICU stay was 12.3 days in our study, similar to the median of 14 days reported in a US study thirty years ago [6] or the median of 13 days reported in a US study more recently [3]. The mean duration of ventilation of 190.3 h in our study is similar to the 8 days reported in a US study [20]. But a study from India showed that the median duration of ventilator was 14 days in a group of patients with MC [21], this result was similar to the previously reported duration of 13 days [22]. We found that pre-intubation PCO_2_ and MG-ADL score at MC onset were associated with duration of ventilation and ICU stay. Higher PCO_2_ prior to mechanical ventilation may indicate more severe condition that will likely require extensive respiratory support and ICU management. Respiratory management is important for MC, one study showed that bilevel positive airway pressure (BiPAP) before the development of hypercapnia was useful in preventing intubation and prolonged ventilation [23]. Another study revealed that hypercapnia at onset predict BiPAP failure and subsequent intubation [24]. Therefore, high PCO_2_ level before intubation may be an independent predictor of prolonged intubation. Thomas et al. identified three risk factors were significantly associated with prolonged intubation, including pre-intubation serum bicarbonate > 30 mg/dl and age > 50 years [3]. In our cohort, the proportion of patients remaining intubated for longer than 2 weeks was 42.9% (21/49) with both hypercapnia and age > 50 years. However, designed prospective study is required verify the statistical significance of various parameters leading to prolonged intubation.

Over 80 % of our patients showed good functional outcome during follow-up. This likely reflects the potentially reversible character of MG and substantial advances in therapeutic and supportive measures [11]. Study has showed that MG is often associated with better functional outcomes at one year than other diseases requiring neurocritical care [25]. However, patients with intermediate and poor outcome had older age of first MC onset, and lower pH and PO_2_, as well as higher PCO_2_ before intubation. Previous study retrospectively included 38 MC patients admitted to the Neuro-medical ICU, and found that 4 patients died in hospital, and the remainder of patients with different age of MC onset (older (> 50 years) and younger (< 50 years) patients) did not show differences in long-term outcome [10]. However, one study found that being older than 50 at first MC independently predicted prolonged intubation [3], while another reported that being younger than 40 at MG onset was associated with higher likelihood of remission [13]. The associations between age of first MC onset and outcomes need to be clarified in larger studies with long follow-up. A study showed that pre-ventilation pH below 7.30 and high PCO_2_ were associated with poor functional outcome and death [26]. A study comparing MC patients in the ward or in the ICU reported that only those in the ICU had abnormal arterial blood gases, and that patients in the ICU had lower pH and higher PCO_2_ [14]. Low pH and high PCO_2_ indicate chronic respiratory acidosis, which may be associated with severe disability and death, especially in MG patients who experience MC. Little is known about the outcome of first MC patients suffering from acute severe exacerbations following ICU discharge. In the present study, pre-intubation PCO_2_ and age of first MC onset were considered to be factors associated with survival. Therefore, in MC patients with extremely high PCO_2_ level before intubation may obtain poorer prognosis, especially in patients with older age.

By the end of follow-up, 21 of 113 patients in our cohort had died (18.6%).This mortality rate is near the high end of the range of 6–30% reported for MC patients in several studies [3, 9, 21, 27, 28]. In a Chinese cohort from Hong Kong, 35 MG patients experienced crisis and 2 died (5.7%) [29], but in a cohort from India, mortality was in 3 out 10 (30%) during MC (30%) [9]. However, the mortality rate of MC fell from 42% in the early 1960s to contemporary rates of 4 to 10% with the improvement of the advent of IVIg and plasma exchange and ICU management [3, 6]. The relatively high mortality rate in our study may reflect the fact that we included all consecutive patients who presented in the neurological ICU during the study period. MC patients were managed in the general ward were not included in the present study. Study has shown that compared to MC patients who received general ward management, MC patients with ICU management had higher MG-ADL scale scores and higher MGFA classification [14]. There could be selection bias, since more seriously ill patients could be selected in the present study. The other fact needed to consider is the ground clinical reality in developing countries, and poor awareness on this part of patients. In addition, drug nonaffordability is the actual reality in China. For example, both plasma exchange and IVIG are not covered by the medical insurance, and the expense on plasma exchange/IVIG is more than the annual income for some Chinese family. In resource-challenged settings like China, vigorous and concerted efforts should be made in MC prevention, timely identification, emergency intervention, and aggressive treatment.

Our study has several shortcomings. First, study have shown that chronic obstructive pulmonary disease (COPD), diabetes mellitus, atrial fibrillation, hyperlipidemia, myocardial infarction, and malignant tumors, were highly associated with death in the MG population [30]. Another study found that at least one comorbid disease was diagnosed 93% patients with late-onset MG (after 60 years) [31]. Our results showed that there was no significant difference among patients with different outcomes regarding the comorbidities, probably due to the small sample size and relatively short follow-up time. Second, our population may have been affected by referral bias because our hospital is a tertiary referral center. With regard to hospital-related factors, it is possible that the apparent benefit of neurological care may be far different from other centers. Some management artefact and treatment strategies may lead to changes in outcomes. As a result of the retrospective design of our study, we may have failed to include certain patients who were not entered properly in the hospital computer system. Third, some patients were unable to receive IVIG or plasma exchange due to the family’s economic hardship. The usage of immunosuppressants in the treatment of MG has greatly changed the outcome of MG patients [32]. One study indicated that azathioprine therapy independently predicted good clinical outcome of MG patients [29]. Another study showed that combined prednisolone-azathioprine treatment reduced the proportion of recurrent MCs, and the number of mechanical ventilation events and ICU admissions were also reduced [33]. Furthermore, the administration of immunosuppressants was found to be closely associated with a decreased risk for death of MG patients [29, 30]. However, only 12 patients received immunosuppressants in the present study. These deficiency in treatment may be associated with the poor prognosis in same patients. Finally, we did not analyze data related to other parameters that might have affected clinical outcomes, including maximal expiratory pressure and maximal inspiratory pressure on pulmonary function tests.

## Conclusions

Despite the limitations of our study, our results clearly show that Higher PCO_2_ prior to mechanical ventilation higher MG-ADL score at MC onset may be useful indicators of whether a patient has more severe or advanced disease that will likely require extensive respiratory support and ICU management. Higher PCO_2_, especially in patients who were older at first MC onset, suggests chronic respiratory acidosis, which may increase risk of severe disability and death. Timely and effective treatment for chronic respiratory acidosis before ICU admission may help prevent exacerbation and improve outcomes.

## Additional file

Additional file 1: **Table S1.** Clinical detail of 115 patients with first MC onset requiring ICU treatment. (DOCX 73 kb)

## Data Availability

The raw data summarized in this article are archived at West China Hospital. Although hospital policy prevents their public dissemination out of concern for patient privacy, individual requests for data access may be granted under appropriate circumstances. Interested parties should contact the authors.

## Abbreviations

AChR: Acetylcholine receptor
AID: Autoimmune disease
BiPAP: Bilevel positive airway pressure
COPD: Chronic obstructive pulmonary disease; yr., year(s)
DM: Diabetic mellitus
h: hour(s)
ICU: Intensive care unit
IVIG: Intravenous immunoglobulin therapy
MC: Myasthenic crisis
MG: Myasthenia gravis
MG-ADL: Myasthenia Gravis–Activities of Daily Living scale
MGFA: Myasthenia Gravis Foundation of America scale
mo: month(s)
MuSK: Muscle-specific kinase
N/A: Not available
NIF: Negative inspiratory force
PE: Plasma exchange
RNS: Repetitive nerve stimulation
SLE: Systemic lupus erythematosus
VC: Vital capacity
WBC: White blood cell count
